# LDH-A regulates the tumor microenvironment via HIF-signaling and modulates the immune response

**DOI:** 10.1371/journal.pone.0203965

**Published:** 2018-09-24

**Authors:** Inna Serganova, Ivan J. Cohen, Kiranmayi Vemuri, Masahiro Shindo, Masatomo Maeda, Mayuresh Mane, Ekaterina Moroz, Raya Khanin, Jaya Satagopan, Jason A. Koutcher, Ronald Blasberg

**Affiliations:** 1 Department of Neurology, Memorial Sloan Kettering Cancer Center, New York, NY, United States of America; 2 Gerstner Sloan Kettering Graduate School of Biomedical Sciences, Memorial Sloan Kettering Cancer Center, New York, NY, United States of America; 3 Bioinformatics Core, Memorial Sloan Kettering Cancer Center, New York, NY, United States of America; 4 Department of Epidemiology and Biostatistics, Memorial Sloan Kettering Cancer Center, New York, NY, United States of America; 5 Department of Radiology, Memorial Sloan Kettering Cancer Center, New York, NY, United States of America; 6 Department of Medical Physics, Memorial Sloan Kettering Cancer Center, New York, NY, United States of America; 7 Department of Medicine, Memorial Sloan Kettering Cancer Center, New York, NY, United States of America; 8 Molecular Pharmacology and Chemistry Program, Memorial Sloan Kettering Cancer Center, New York, NY, United States of America; University of South Alabama Mitchell Cancer Institute, UNITED STATES

## Abstract

Previous studies show that LDH-A knockdown reduces orthotopic 4T1 breast tumor lactate and delays tumor growth and the development of metastases in nude mice. Here, we report significant changes in the tumor microenvironment (TME) and a more robust anti-tumor response in immune competent BALB/c mice. 4T1 murine breast cancer cells were transfected with shRNA plasmids directed against LDH-A (KD) or a scrambled control plasmid (NC). Cells were also transduced with dual luciferase-based reporter systems to monitor HIF-1 activity and the development of metastases by bioluminescence imaging, using HRE-sensitive and constitutive promoters, respectively. The growth and metastatic profile of orthotopic 4T1 tumors developed from these cell lines were compared and a primary tumor resection model was studied to simulate the clinical management of breast cancer. Primary tumor growth, metastasis formation and TME phenotype were significantly different in LDH-A KD tumors compared with controls. In LDH-A KD cells, HIF-1 activity, hexokinase 1 and 2 expression and VEGF secretion were reduced. Differences in the TME included lower HIF-1α expression that correlated with lower vascularity and pimonidazole staining, higher infiltration of CD3^+^ and CD4^+^ T cells and less infiltration of TAMs. These changes resulted in a greater delay in metastases formation and 40% long-term survivors (>20 weeks) in the LDH-A KD cohort following surgical resection of the primary tumor. We show for the first time that LDH-depletion inhibits the formation of metastases and prolongs survival of mice through changes in tumor microenvironment that modulate the immune response. We attribute these effects to diminished HIF-1 activity, vascularization, necrosis formation and immune suppression in immune competent animals. Gene-expression analyses from four human breast cancer datasets are consistent with these results, and further demonstrate the link between glycolysis and immune suppression in breast cancer.

## Introduction

Lactate dehydrogenase A (LDH-A) is required for the maintenance and progression of many tumors [1–4], and inhibition of LDH-A has an anti-proliferative effect [2, 5–11]. Nevertheless, a detailed understanding of how LDH-A facilitates immune suppression and tumor progression is not fully understood. Given the importance of glycolytic metabolism and its metabolites on the immune response [12, 13] and on tumor progression [2, 14–17], and the effect of lactate on the activation of Hypoxia-Inducible Factor 1 alpha (HIF-1α) [18–20], we have explored the effect of LDH-A knockdown on HIF-1 responses by introducing HIF-1 reporters into tumor cells. HIF-1 binds to more than 1,000 genes and activates genes involved in metabolic reprogramming [21, 22], including upregulation of a glucose transporter (GLUT1), hexokinases (HK1 and HK2), and the lactate-secreting monocarboxylate transporter 4 (MCT4). High lactate levels stimulate angiogenesis [23, 24] through activation of the VEGF/VEGFR2 signaling pathway [19, 25, 26], in addition to HIF-mediated regulation.

HIF-signaling impacts tumor cell invasion and migration from the primary site [27]. Furthermore, tumor hypoxia and high HIF-1 activity promote an immunosuppressive phenotype (involving both tumor cells and infiltrating immune cells) that has a direct effect on metastatic tumor progression [27]. Understanding the mechanisms by which HIF-signaling promotes immunosuppression is under investigation and may have important therapeutic implications in the treatment of metastatic disease [28, 29].

In this study, we used an orthotopic, highly immunogenic and metastatic murine breast carcinoma model (4T1) [30] in order to determine: i) whether LDH-A depletion and the resulting metabolic alterations would change the tumor microenvironment (TME); ii) whether LDH-A/lactate modification impacts on HIF-1 signaling and its downstream targets; and iii) how these alterations affect the anti-tumor immune response and the development of metastases in immunocompetent mice. We also compare these results with a gene-expression analysis from a compendium of four human breast cancer datasets, and show a clear association between high LDH-A and HIF-1α expression and poor clinical outcome (metastases-free survival).

## Materials and methods

### Cell lines and reagents

4T1 cells derived from a spontaneous breast tumor in a BALB/c mouse (provided by Dr. Fred Miller; Karmanos Cancer Institute) were studied [31]. All modified derivatives from 4T1 murine breast cancer cells, were grown in DMEM containing 10% FCS supplemented with 25 mM glucose and 6 mM L-glutamine, penicillin/streptomycin, with the addition of 500 mg/L G418 and 4 mg/L of puromycin.

We constructed a new bioluminescence HRE-reporter vector, where the HSV1-tk/GFP/FLuc [32] fusion reporter was substituted for the exGLuc-IRES2-GFP cassette [33]. A new SFG-tdRFP/FLuc retroviral vector was kindly provided by Dr. Vladimir Ponomarev [34]. All retroviral plasmids were transfected into the GPG29 packaging cell line with Lipofectamine 2000 (Invitrogen, Carlsbad, CA, USA). The retrovirus-containing medium was collected and stored at -80°C. First, 4T1 cells were transduced by incubating 50% confluent cells with dxHRE-exGLuc-IRES2-GFP-Neo virus-containing medium for 12 h in presence of polybrene (8 μg/ml; Sigma). Selection of stably transduced cells was accomplished by adding 500 mg/L of G418 to cells transduced with vector. Cells containing the dxHRE-exGLuc-IRES2-GFP-Neo reporters system were further transduced with a second retroviral vector SFG-tdRFP/FLuc. Subsequently cells were sorted using GFP or tdRFP as fluorescence markers and validated for reporters activity (**Panels A and B in S1 Fig**).

### Generation of LDH-A knockdown and control cell lines

4T1-HREexGLuc-IRES2-GFP-Neo/tdRFP/FLuc positive cells were transfected with SureSilencing™ shRNA plasmids (QIAGEN, Frederick, MD, USA) to specifically knockdown expression of the mouse *LDH-A* gene as described previously [11]. Stably transfected clones (LDH-A KD cell lines) were developed, as described previously [11]. LDH enzyme and LDH-A immunoblotting protein assays confirmed successful transfection and LDH-A shRNA knock-down in different clones showing different degrees of LDH-A expression (**Panels A and B in S2 Fig**).

### *In vitro* bioluminescence assays

Stably transduced and sorted cells were seeded in 6-well plates. The medium was changed 24 h later to fresh medium containing CoCl_2_ (100 μM) (Sigma-Aldrich, St. Louis, MO, USA). Alternatively, cells were incubated under hypoxic conditions (1% O_2_) for 6 and 24 h. The cells were collected in media with 10% FCS, counted with a disposable hemocytometer (Invitrogen, Carlsbad, CA, USA), and assessed for viability by a trypan blue staining. An IVIS Spectrum In Vivo Imaging System (PerkinElmer, Caliper Life Sciences, Mountain View, CA) was used to measure Firefly Luciferase (FLuc) and exGaussia Luciferase (exGLuc) activities. The acquisition time was dependent on the signal intensity in the different reporter cell lines. All measurements are reported as photons/second/2x10^4^ cells. Bioluminescence assays were always performed with 2 x 10^4^ cells in 96-well plates using 10 μl of Bright-Glo Luciferase solution (Promega Cor., Madison, WI, USA) or luciferin (30 mg/ml) for Firefly Luciferse (FLuc) or 5 μl of a water-soluble Coelenterazine (NanoLight Technology, Pinetop, AZ, USA) (5 μg) for exGLuc.

### Western blotting

Cell lines underwent protein extraction using RIPA buffer (Thermo Fisher Scientific, Waltham, MA, USA). Protein concentrations were determined by Pierce BCA protein assay (Thermo Fisher Scientific, Waltham, MA, USA). The proteins in equivalent amounts (10–40 μg/well) were separated by electrophoresis in a NuPAGE gradient 4–12% Bis-Tris Gel (Invitrogen, Carlsbad, CA, USA) and were immuno-blotted with anti-LDH-A antibody (Cell Signaling Technology, Danvers, MA, USA) at a 1:1,000 dilution and anti-ß-actin antibody (Abcam Inc., Cambridge, MA, USA) at a 1:5,000 dilution antibodies. In addition, we used Hexokinase I and II (Cell Signaling Technology Inc., Danvers, MA, USA) to detect the expression of these glycolytic enzymes as described by the protocol. Immune complexes were detected by horseradish-peroxidase-labeled antibodies and enhanced chemiluminescence reagent (Amersham, Buckinghamshire, UK).

### ELISA for murine VEGF-A

The amount of VEGF-A in the culture medium was determined using the Quantikine ELISA kit for mouse VEGF (R&D Systems, Minneapolis, MN, USA). VEGF-A ELISA was conducted according to the manufacturer's instructions. Supernatants from 4T1-HRE reporter cells exposed to 21% O_2_, 1% O_2_ and 100 μM CoCl_2_ for 6 or 24 hours were harvested. Additionally, 4T1-HRE reporters cells were incubated under different conditions: addition of 30 mM lactic acid, 30 mM Sodium Lactate (Na-Lactate) for 6 and 24 hours, after which cell culture supernatants were harvested and preserved and a number of cells were counted. The data was normalized to total cell counts.

### ddPCR

For RNA purification, cells were grown for 48 hours (exponential growth phase). RNA was isolated using the RNeasy total RNA isolation kit (Qiagen), following the manufacturer’s protocol. Quantitative digital droplet PCR (ddPCR) for LDH-A, MCT1 (*Slc16a1*-Monocarboxylate transporter 1), MCT4 (*Slc16a3—*Monocarboxylate transporter 4) and β-actin was performed by the Genomics Core Lab at MSKCC.

### LDH—Activity

Total LDH activity was assessed using the Cytotoxicity Detection Kit PLUS (LDH) (Roche Diagnostics,Florham Park, NJ). Different numbers of cells were plated in 96-well plates and incubated (37°C, 5%CO_2_, humidified incubator) for 2 hours for their attachment. LDH activity from lysed cells was measured as described [14].

### Metabolic extracellular flux analysis

Glycolytic activity of cells was measured using a Seahorse XF96 Extracellular Flux Analyzer (Agilent Seahorse XF Technology, Billerica, MA, USA). Cells were seeded at 25,000–30,000 cells per well in Seahorse XF96 96-well plates and allowed to attach overnight in a 37°C incubator under 95% air/5% CO_2_, in a standard growth media. DME medium without phenol red with 2 mM glutamine, 10 mM glucose, 1 mM pyruvate and 5 mM HEPES (pH 7.4) was used as running medium in the XF assays and is referred to as “assay medium.” Assays were initiated by removing the growth medium from each well and replacing it with 180 μl of the Seahorse assay medium pre-warmed to 37°C. The cells were incubated at 37°C for 60 min to allow media temperature and pH to reach equilibrium before the measurement. After this time period, media was replaced with a fresh assay media to remove background. The Proton Efflux Rate (PER, largely reflecting glycolysis) and the oxygen consumption rate (OCR, largely reflecting oxidative phosphorylation) were measured simultaneously for 4 min to establish a baseline rate. The assay medium was then gently mixed again for 2 min between each measurement to restore normal oxygen tension and pH in the microenvironment surrounding the cells. This was repeated four times. After the baseline measurements, 20 μl of a test agent, (5 μM Rotenone/Antimycin A (Rot/AA)), was then injected into each well to reach the desired final working concentration (0.5 μM Rot/AA). Measurements were repeated four more times, after which 22 μl of a second test agent, 500 mM 2-deoxy glucose (2-DG; final working concentration 50 mM) was injected, followed by 4 independent measurements. Total PER was measured by plotting the proton efflux as a function of time (pmol/min). OCR was measured as the oxygen tension of the media as a function of time (pmol/min). Data was normalized to the total protein amount in each individual well.

### *In vivo* bioluminescence imaging (BLI)

The animal protocol was approved by the Memorial Sloan-Kettering Institutional Animal Care and Use Committee (IACUC). The 4T1-HRE-exGLuc-IRES-GFP-Neo/tdRFP/Fluc cells bearing a scramble shRNA (A5NC) and specific for LDH-A shRNA A2-10KD and A3-8KD (1x10^6^) were injected subcutaneously into the 4^th^ mammary fat pad of 4-6-weeks old female BALB/cAnN mice (Charles River Lab, USA). BLI was performed during the first 3–14 days for HIF-1 activity using exGLuc in the primary tumors with a water-soluble Coelenterazine (NanoLight Technology, Pinetop, AZ, USA) (50 μg) and for FLuc activity each week during experiments for metastatic development with 50 μl of D-Luciferin (30 mg/ml) (Gold Biotechnology, St. Louis, USA). Photons emitted from the tumor region were quantified using Living Image software (PerkinElmer, Caliper Life Sciences, Mountain View, CA). The BLI HIF-1 reporter data was normalized to the baseline (week 1) value. Animal number: 4T1-HRE A5NC n = 4, LDH-A A2-10KD n = 7 and A3-8KD n = 7; error bars: SEM instead SD.

In order to model the clinical management of breast cancer, the primary tumors were surgically excised. Utilizing a technique detailed in the IACUC animal protocol (# 08-07-011), the tumor mass with a draining lymph node was removed and any blood vessels leading into the tumor were cauterized. The incision was closed using interrupted non-absorbable monofilament suture or sterilized wound clips (Autoclips). Mice were checked daily and BLI was performed once per week to detect distant-site metastatic nodules, and recurrence of the primary tumor. Mice were sacrifice if they become moribund, according to IACUC guidelines.

### Immunohistochemical staining

Dissected tumors were immediately placed into 4% paraformaldehyde for further immunohistochemistry. The immunofluorescent staining was performed at Molecular Cytology Core Facility of MSKCC using Discovery XT processor (Ventana Medical Systems). 5 μm thick, paraffin-embedded sections were stained for H&E to detect necrotic areas. Adjacent sections were stained with antibodies for CD31 (endothelial cell marker), CD3 (T cells), CD4 (T cells), F4/80 (TAMs) and HIF-1α (1 μg/ml, cat# NB100-654, (Novus Bio., CO)). Analysis of blood vessel density and immunofluorescence against CD3^+^, CD4^+^, CD31 and HIF-1α was performed and quantified using MetaMorph software by thresholding images and counting the number of T-cells or TAMs /mm^2^ and % area covered by CD31 or HIF-1α as described previously [35]. For intratumoral analysis of T-cells and vessels, tumors were divided into ~400 μm^2^ squares and the percentage of CD3-, CD4- or CD31-positive pixels was calculated per tumor square and plotted. Several animals were injected with Pimonidazole hydrochloride (hypoxyprobe-1, HPI) at 40 mg/kg. Pimomidazole hydrochloride was administered one hour pre-sacrifice [36].

### Analysis of breast cancer microarray datasets

A compendium of four breast cancer microarray datasets was analyzed using the Bioconductor set of tools (www.bioconductor.org) in R statistical language (www.r-project.org). Data was downloaded from GEO. Four breast cancer datasets, profiled on the HG_U133 Affymetrix platform, were analyzed: A) MSKCC-82 GSE-2603 [36], B) EMC-286 GSE-2034 [37], C) ECM 192 GSE12276: 204 samples [38], and D) EMC-344 (EMC 286 AND 58 cases of ER- tumors, GSE 5327)[39]. Three datasets are on the HG-133A platform (one group) and one dataset is on the HG133 Plus2 platform (another group). All datasets were normalized using the standard gcrma procedure [40] and then the data for common probes were combined into one dataset. Values for multiple probes that correspond to the same genes were averaged. Values for LDH-A and HIF-1α were averaged for each patient, patients were separated into thirds based on their average expression of LDH-A & HIF-1α (Low, Medium, High), and their metastasis-free survival was plotted as a measure of time. Additionally, Cox proportional hazard ratios were calculated between the Low vs. High groups and these Hazard Ratios and p-values were plotted. The same was done for CD3E, CD8A and CD4. For the Combined CD3E, CD4, CD8A, LDH-A and HIF-1α plots, the Average LDH-A & HIF1α expression value was subtracted from the Average CD3E, CD4 & CD8A expression value on a per patient basis, and the patients were then divided into thirds as before according to their expression of the new “Combined” expression (Low, Medium, High). Thus, “Low” expression of this combined value represents patients with low expression of CD3E, CD4 & CD8A and high expression of LDH-A & HIF-1α, while “High” expression of this combined value represents patients with high expression of CD3E, CD4 & CD8A and low expression of LDH-A & HIF1α.

### Statistical analysis

Results are presented as mean ± standard deviation, where relevant. Tumor growth profiles were analyzed by fitting a linear model to log-transformed tumor volume data with main effects for date (since experiments were done on two different dates), animal type and time and a random effect (since each animal was measured at multiple time points). Statistical significance of the main effects was determined via a two-sided t-test using a p-value of < 0.05 to declare significance.

## Results

### Characterization of LDH-A knock-down 4T1 cells bearing constitutive and HRE-sensitive reporters for bioluminescence imaging (BLI)

A new set of LDH-A knock-down cells was developed, for comparison with our previous published results [11]. These dual-reporter cells were used to monitor tumor progression as well as HIF-1 activity by BLI. First, 4T1 cells were transduced with a retroviral vector, where a hypoxia response element (HRE) [37] drives the expression of exGLuc [38] and GFP **(Panel A in S1 Fig**)[33, 37, 39]. Second, to visualize the development of metastasis, selected and enriched populations of 4T1-HRE-exGLuc-IRES2-GFP-Neo cells were used for a second transduction, where a retroviral vector containing a tdRFP/Fluc fusion gene was placed under a constitutive LTR promoter (**Panel A in S1 Fig**). The activity of the bioluminescence reporters was evaluated (**Panel B in S1 Fig**) in the developed parental cell line (4T1-HRE-exGLuc-IRES2-GFP-Neo/tdRFP-FLuc).

To assess the connection between LDH-A expression and the effect of LDH-A expression on metabolic changes, including HIF-1 pathway activity, we transfected 4T1-HRE-exGLuc-IRES2-GFP-Neo/tdRFP-FLuc parental cells with SureSilencing shRNAs plasmids, specifically targeting mouse LDH-A mRNA (KD). To establish a control cell line (A5NC), the same parental cells were transduced with a non-specific scrambled shRNA, as described previously [11]. Several knock-down clones with different levels of LDH-A protein expression (as assessed by immunoblotting) were isolated for further experiments. Two clones (A2-10KD, A3-8KD) with different levels of LDH-A expression were chosen for further work (**Panel A in S2 Fig**). *In vitro* growth assays demonstrated slow growth for both the A2-10KD and A3-8KD clones, compared to control A5NC cells (**Panel C in S2 Fig**). To validate the correlation between LDH-A expression levels and functional activity of the LDH enzyme complex, we performed an enzymatic assay to assess LDH activity. A reduction of 60% and 20% of LDH activity was observed in 4T1-HRE A2-10KD and 4T1-HRE A3-8KD cells, respectively, compared to 4T1-HRE A5NC cells (**Panel B in S2 Fig**).

Glycolytic rates in the developed cell lines were measured (Agilent Seahorse Bioscience XF96 Extracellular Flux Analyzer)(**Panel D and E in S2 Fig**). A significant difference was detected in compensatory glycolysis following Rotenone/Antimycin injection between 4T1-HRE-A5NC and the LDH-A knockdown cells. Following LDH-A knock-down, both 4T1-HRE-A2-10KD and 4T1-HREA3-8KD cells showed less dependence on glycolysis than control 4T1 NC cells, despite the different levels of LDH-A enzyme activity and expression on Western blot between the two KD cell lines.

### Tumor growth and development of metastasis in immunocompetent mice

**Fig 1:**
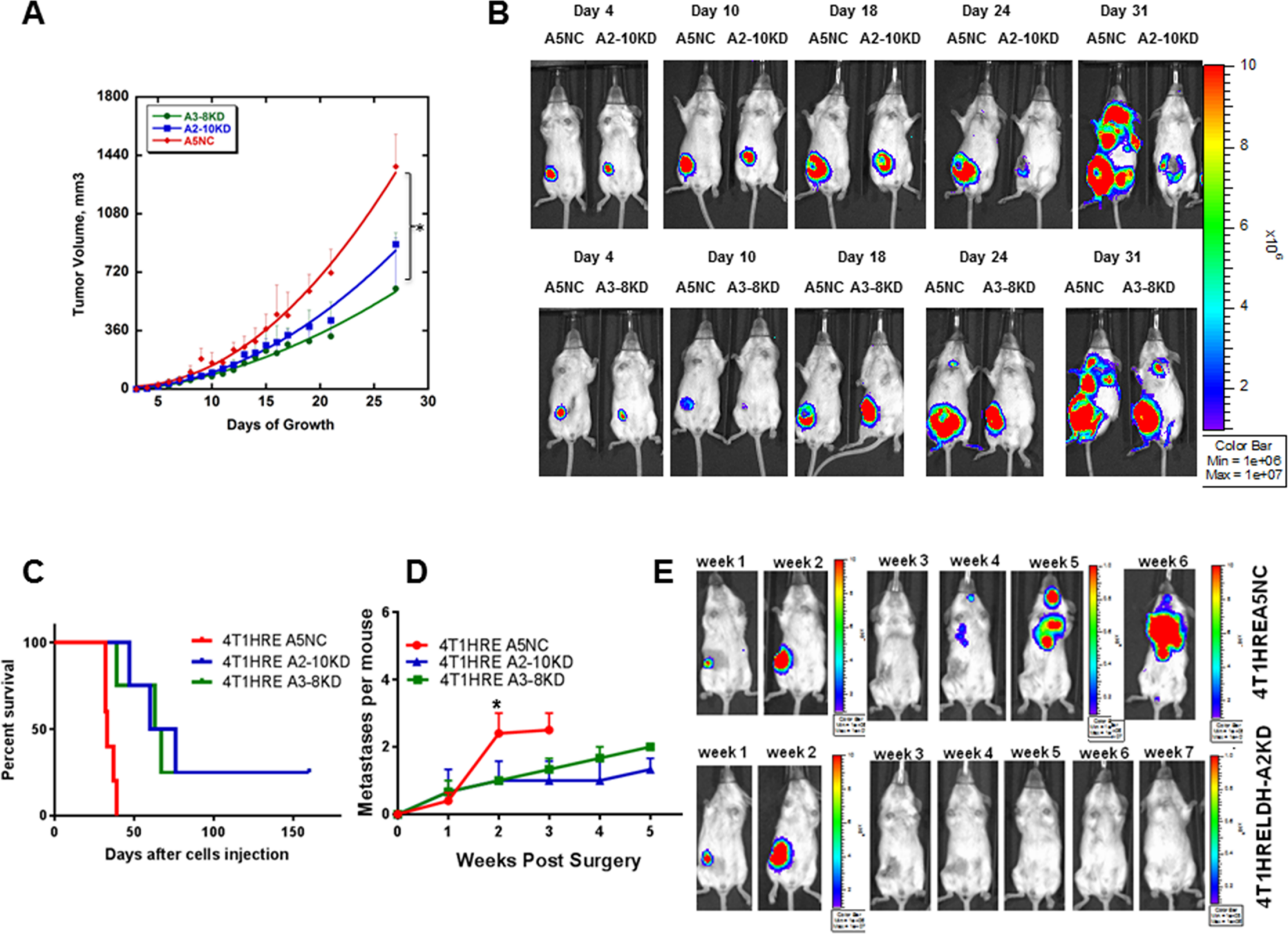
Effect of LDH-A knockdown on primary tumor growth and development of metastases. Comparison of control (4T1-HRE A5NC) and LDH-A knock-down (4T1-HRE A2-10KD, 4T1-HRE A3-8KD) tumor growth in immunocompetent BALB/c mice. Mice were injected with 1.0 × 10^6^ tumor cells into the 4th mammary fat pad; tumor growth was monitored by caliper measurements (A) and by Firefly luciferase/luciferin bioluminescence imaging (BLI) (B). LDH-A knockdown delayed the growth of primary tumors, compared to 4T1-HRE A5NC control tumors. Statistcs: p<0.005 (A). Metastatic free survival in immune competent BALB/c mice bearing control (NC) and LDH-A knock-down (KD) tumors were compared. Survival was determined from the day of orthotopic tumor cell implantation, and surgical resection of the primary tumor. Surgical resection was performed 9–13 days after tumor cell inoculation when the size of the primary tumor was 100–200 mm^3^ (C). Firefly luciferase/luciferin BLI was used to detect metastasis after surgical resection of the primary tumor (E). A comparison of the number of metastases/mouse observed in the BLI images of BALB/c mice bearing KD and NC tumors; significance (p<0.05) was observed at week 2 (D). The analysis of KD tumor-bearing animals was limited to animals that developed metastases and did not survive during the 160 day observation period.

Previously, we reported that downregulation of LDH-A expression in 4T1 murine breast cancer cells leads to slower growth, reduced glycolytic flux and increased mitochondrial respiration, leading to the delayed onset of distant metastases in immunocompromised mice [11]. We performed similar experiments in immunocompetent mice using two different BLI reporters; i) an HRE-responsive exGLuc as a readout of HIF-1 activity, and ii) a constitutive FLuc as a readout of metastasis formation. The inhibitory effect of LDH-A depletion on the tumor growth was significant (p = 0.0002 comparing A3-8KD vs. A5NC; p = 0.0005 comparing A2-10KD vs A5NC (**Fig 1A**). There was no significant difference in the tumor volume profiles between A3-8KD and A2-10KD (p = 0.50). BLI for FLuc (constitutive expression) showed a similar growth pattern in all 3 sets of tumors and was previously described [40]. The development of metastases was delayed in mice bearing LDH-A knock-down tumors, compared with mice bearing NC primary tumors. All animals bearing orthotopic A5NC tumors developed metastases by week 4 after tumor inoculation. No animals bearing orthotopic LDH-A KD tumors developed visible metastases over the first 3 weeks (**Fig 1B**). At later time points, a significant increase in total BLI signal was observed in mice bearing 4T1-HRE A5NC tumors compared to LDH-A KD tumors (e.g., Day 31, **Fig 1B**).

To more closely simulate the clinical management of breast cancer (where the primary tumor is removed), a separate group of animals underwent surgical resection of the primary mammary tumor at 9–13 days after tumor cell inoculation when the size of the primary tumor was 100–200 mm^3^ followed by weekly FLuc BLI to observe the development of metastasis. In cohorts where the primary tumor was resected, mice initially bearing orthotopic LDH-A KD tumors developed significantly fewer metastases and survived longer than mice initially bearing control NC tumors (**Fig 1C, 1D and 1E**). All animals in the A5NC group died from metastatic disease by 40 days (**Fig 1C**). In contrast, animals in the LDH-A KD group started to die later, at 49 days (**Fig 1C**). Importantly, survival was markedly extended for 40% of these mice; they remained alive for over 20 weeks. Furthermore, for the KD tumor-bearing mice that did not survive, there were significantly fewer metastases visualized by BLI compared to the NC tumor-bearing animals (p<0.0001 for A2-10KD and p = 0.001 for A3-8KD vs. A5NC)(**Fig 1D**).

### Immunofluorescence staining of host cells within the tumor microenvironment

**Fig 2:**
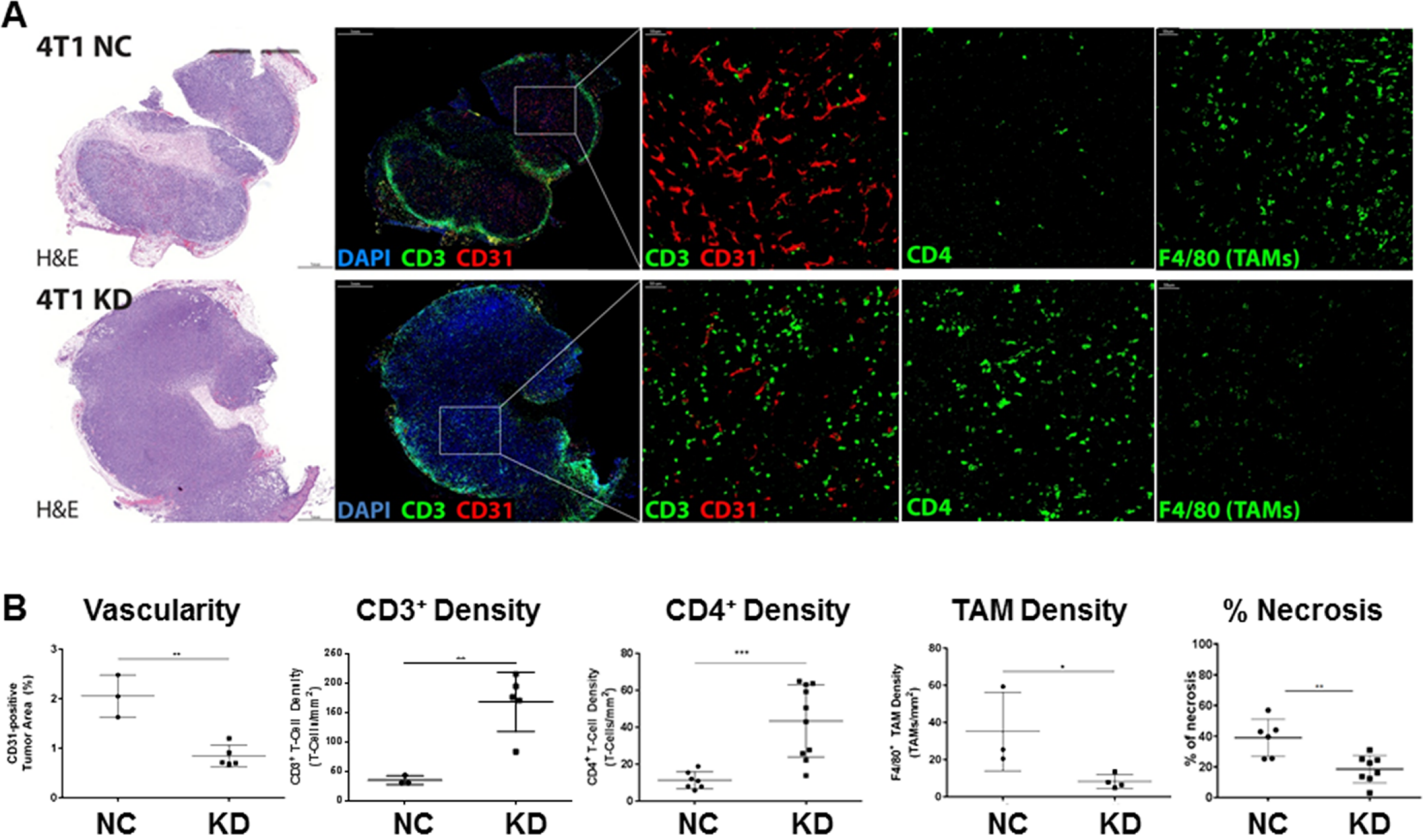
Effects of LDH-A depletion on intra-tumoral host cells, CD3^+^, CD4^+^ T cells, CD31^+^ blood vessels, F4/80+ TAMs, necrosis. H&E staining and immunofluorescence (IF) imaging of CD31^+^ vessels, CD3^+^ T cells, CD4^+^ T cells and TAMs (using F4/80 as a macrophage marker); scale bar, 100 μm (**A**). Quantitative analysis of blood vessel density (% area covered by CD31 staining), and density of CD3^+^ and CD4^+^ T cells and F4/80^+^ TAMs density using MetaMorph software (thresholding images) and counting the number of T-cells or TAMs (#/mm^2^). Statistics: *p< 0.05; **p<0.01, ***p< 0.001, (**B**). H&E staining was performed. The extent of necrosis was assessed using ImageJ. Statistics: **p<0.01 (**A**).

Following surgical resection of primary tumors, sectioning and staining for H&E, CD31, CD3, CD4, F4/80, HIF-1α and pimonidazole was performed and quantified using MetaMorph software [35]. 4T1 tumors start developing necrosis at volumes greater than ~100 mm^3^, and necrosis increases significantly as tumors grow beyond 200–300 mm^3^ [14]. Quantification of the necrotic area on H&E sections revealed that A5NC tumors had significantly more necrosis compared to LDH-A KD tumors (39.1±12.1% vs. 18.5±8.9%, respectively; (p<0.01))(**Fig 2A and 2B**).

Immunofluorescence staining (IF) for CD3^+^ and CD4^+^ T cells showed that they were excluded from the central part of A5NC tumors and were restricted to the periphery (**Fig 2A and 2B** and **Panels A and B in S3 Fig**). In contrast, LDH-A-depleted tumors showed a significantly greater number of CD3^+^ and CD4^+^ T-cells within the interior (4.8 and 3.3-fold increase, respectively, compared to A5NC tumors) (**Fig 2A and 2B** and **Panels A and B in S3 Fig**). We also noticed that CD3^+^ T cell size increased in LDH-A knock-down tumors, from 51±4 μm^2^ (A5NC) to 61±2 μm^2^ (LDH-A KD) (p<0.0001) (**Panel C in S3 Fig**).

The opposite pattern was observed for F4/80+ Tumor Associated Macrophages (TAMs): more TAMs were detected in A5NC tumors compared with LDH-A knock-down tumors (4.3 fold differences (**Fig 2A and 2B**). To evaluate the degree of tumor neovascularization, we stained tumors for CD31 and quantified the area of tumors covered by CD31+ vessels as a percentage of total tumor area. CD31 staining revealed substantially lower formation of new blood vessels in LDH-A KD tumors by percentage (**Fig 2B**). Detailed analysis of intratumoral distributions of T-cells (CD3) and vessels (CD31) revealed a negative correlation between these parameters (**Panel D in S3 Fig**).

### LDH-A down-regulation impacts the HIF-1 pathway

Lactate and pyruvate regulate hypoxia-inducible gene expression, independent of physical hypoxia, by increasing the accumulation of HIF-1α [18]. Augmenting the DNA binding activity, HIF-1 enhances the expression of several HIF-1-activated genes, including erythropoietin, vascular endothelial growth factor (VEGF), glucose transporter 3, and aldolase A [18, 19]. Based on these observations, the effect of LDH-A knock-down on the activity of the HIF-1 reporter was assessed in the 4T1-HRE cell lines.

**Fig 3:**
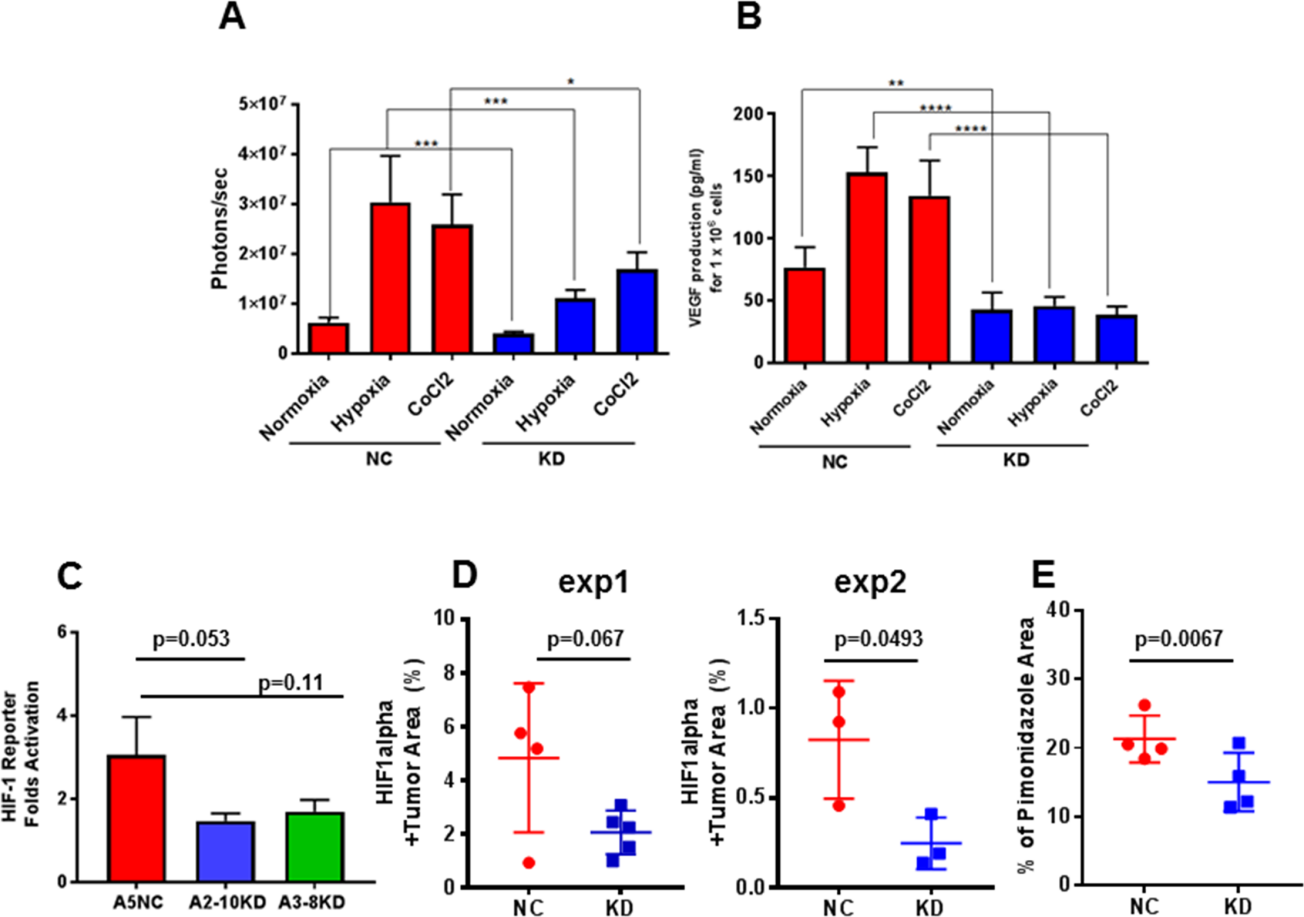
The impact of LDH-A inhibition on HIF-1 activity. The assessment of HIF-1 activity after 6 hours of different treatments (**A**). VEGF-A secretion (a downstream target of HIF-1) by 4T1-HRE A5NC and A2-10KD cells under different conditions over 6 hours (**B**). The BLI-based HIF-1 and ELISA-based VEGF-A measurements are normalized to cell number. Statistics: **** p<0.0001; *** p = 0.0002; **p = 0.0071; *p< 0.05. **HIF-1 activity in tumors (C-E).** Bioluminescence imaging of HIF-1 reporter activity in 4T1-HREexGLuc-IRES2-GFP tumors was performed at week 1–2. Data are expressed as tumor-to-background ratios normalized to initial values of week 1; ±SEM (**C**). Analyses of percent HIF-1α positive cells and area of Pimonidazole staining were performed and quantified using MetaMorph software by thresholding images. A greater percent of HIF-1α positive cells (**D**) and area of pimonidazole staining are seen in 4T1-HRE A5NC tumors compared to the LDH-A knock-down 4T1-HRE A2-10KD and 4T1-HRE A3-8KD tumors (**E**). Two independent experiments were performed, and immunofluorescence staining was performed on different days and on different samples.

First, the basal level of HIF-1 reporter readout (HRE-exGluc BLI) was observed to be lower in LDH-A KD cells, compared to A5NC cells (**Fig 3A**). Second, exposure of cells to hypoxia (1% O_2_) or cobalt chloride (100 μM CoCl_2_, a hypoxia mimetic) for 6 hours showed a significantly lower effect on HIF-1 upregulation in LDH-A knock-down cells (KD) compared to A5NC control cells (**Fig 3A**). Third, VEGF-A secreted by cells in the above experiments were measured by ELISA, and observed to be significantly lower in media from LDH-A KD cells compared to A5NC cells, under both normoxic and hypoxic conditions (**Fig 3B).** Fourth, the levels of HK1 and HK2 expression were assessed in cell lysates and were found to be higher in A5NC cells compared with LDH-A KD cells under standard growth conditions (**Panel A in S4 Fig**). We also determined the mRNA expression levels of the monocarboxylate (lactate, pyruvate) transporters 1 (MCT1) and 4 (MCT4), correspondingly. Interestingly, a lower, almost non-detectable expression level of MCT4 was observed (**Panel B in S4 Fig**). In contrast to it, MCT1 expression level was significantly higher by 180-fold. However, no differences in MCT1 expression were seen between A5NC and LDH-A KD cell. As expected, there was a marked decrease in LDH-A expression seen in LDH-A KD cells (**Panel B in S4 Fig**).

### LDH-A downregulation effects on HIF-1 expression in tumors

To determine whether LDH-A inhibition affects HIF-1 activity *in vivo*, BLI intensity of the HRE-exGLuc reporter (HIF-1 readout) was compared in A5NC and LDH-A KD tumors at two different times (after 1 and 2 weeks of tumor growth) (**Fig 3C**). A ~3-fold increase in exGLuc expression (HIF-1 reporter) was observed in A5NC tumors, whereas only a 1.5–1.7 fold increase was observed in the LDH-A KD tumors (**Fig 3C**). To confirm that changes in the bioluminescence signal reflected changes in HIF-1α, tumor sections were stained for HIF-1α IF. LDH-A KD tumors showed considerably less of HIF-1α staining in two independent experiments (51.7α0.6% of A5NC) (**Fig 3D**). In addition, we also collected tumor samples after administration of Pimonidazole hydrochloride (hypoxia probe). Consistent with the HIF-1α staining and HRE-reporter data, A5NC tumors demonstrate greater pimonidazole (hypoxia) staining than LDH-A KD tumors (**Fig 3E**).

### Lactate stimulates HIF-1 pathway

**Fig 4:**
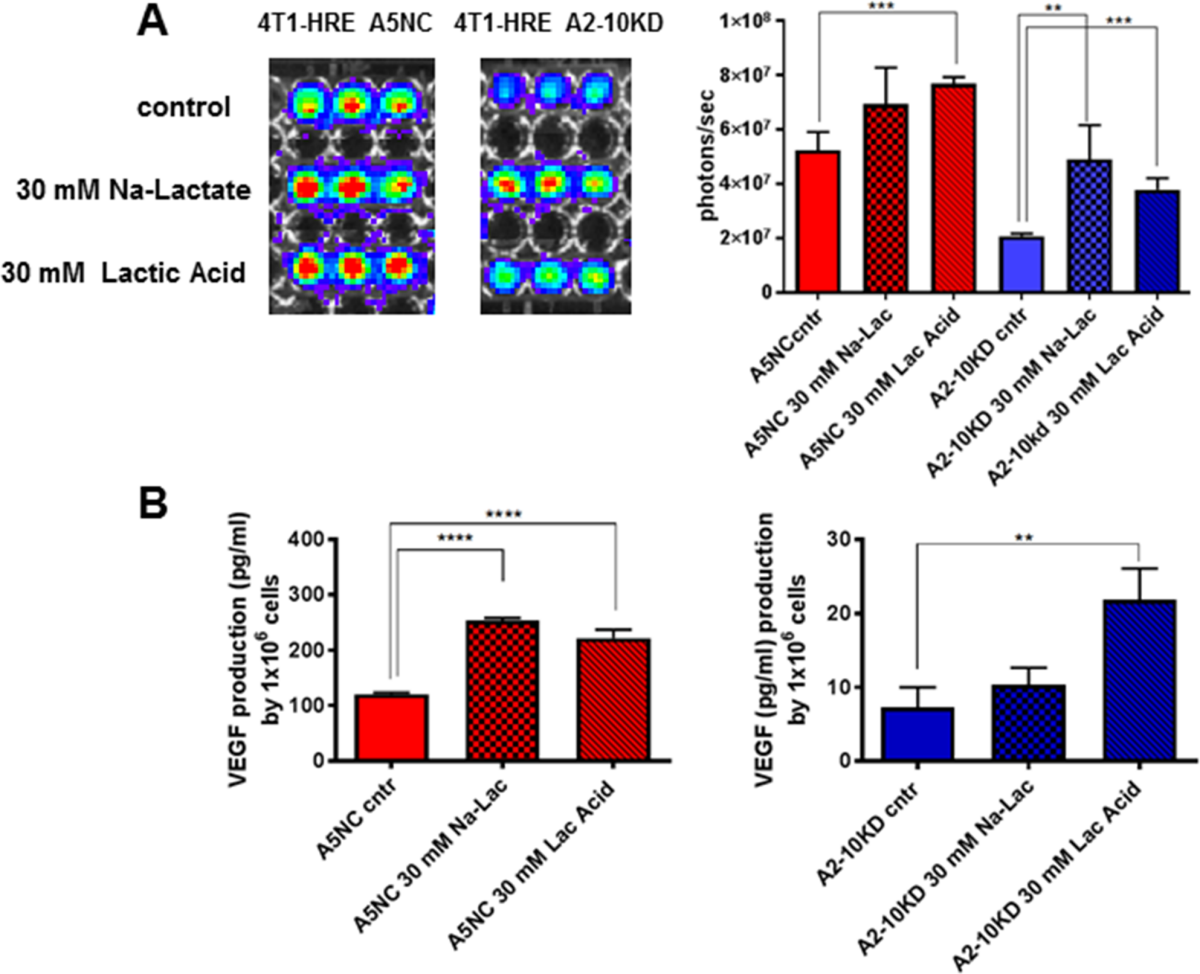
Na-lactate and Lactic Acid effects on HIF-1 and VEGF-A, in LDH-A KD and control NC cells. Assessment of HIF-1 activation using the HRE reporter system (**A**) and its downstream target—*VEGF165* (**B**). Measurements followed a 24 h incubation in 30 mM Lactic Acid or 30 mM Na-Lactate. Statistics: *p<0.05; **p<0.01; ***p<0.005; ****p<0.001.

Previous studies have shown that lactate can lead to an increase in the level of HIF-1α [18, 19]. To test whether Lactate induces HIF-1 activation, we added Na-Lactate or Lactic Acid to the media (30 mM) for 24 hours, and than quantified HRE-exGLuc reporter activity (BLI) (**Fig 4A**). We also detected a small increase of VEGF-A secretion in 4T1-HRE A5NC and A2-10KD cells under the same conditions (**Fig 4B**). These experiments showed that addition of Na-Lactate or Lactic Acid to the media for 24 hrs is sufficient to cause an increase in HIF-1 activity and VEGF-A secretion *in vitro*.

### Increased metastatic propensity of LDH-A KD cells in the presence of exogenous lactate

To determine whether the addition of exogenous Na-Lactate would affect the initial growth of tumors and the development of metastasis, 4T1-HRE A5NC or LDH-A KD tumor cells were mixed with Matrigel in the presence or absence of 30 mM Na-Lactate (Na-Lactate matrigel plug) [19]. In the first set of mice (n = 5/group) the growth of primary tumors was assessed. At 21 days, the volume of the A2-10KD clone with the addition of Na-Lactate to the Matrigel plug was slightly larger than the lactate-free group (960±50 vs 634±107 mm^3^, but not statistically significant; p = 0.1)(**Panel A in S5 Fig**). A similar trend was detected for A3-8KD group (1042±321 and 888±257 mm^3^, respectively (**Panel B in S5 Fig**)).

**Fig 5:**
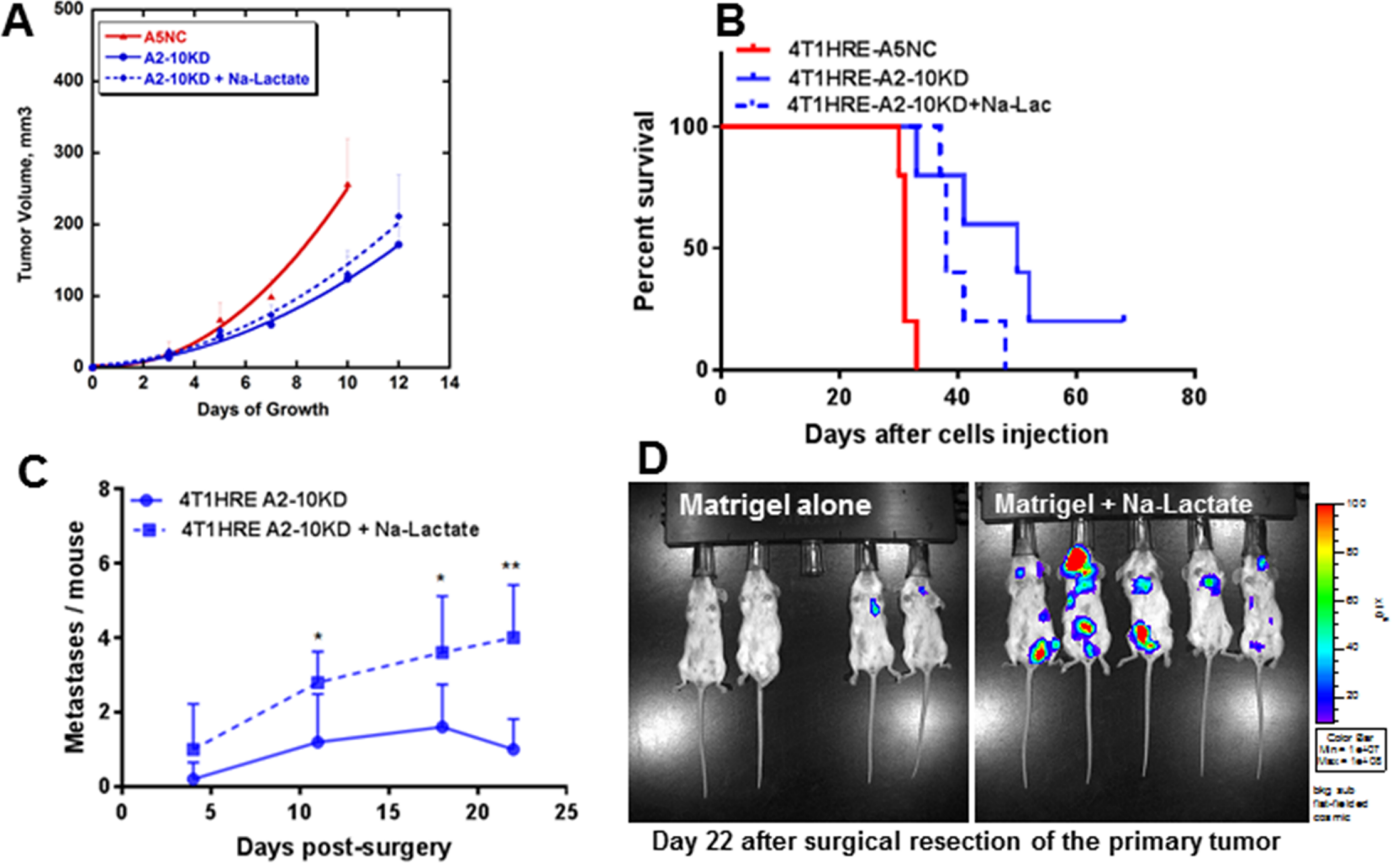
Lactate effect (matrigel plug) on LDH-A KD tumor growth, development of metastases and survival. Tumor growth profiles following orthotopic injection of 2x10^5^ 4T1-HRE A5NC and A2-10KD cells in 50% of standard Matrigel and in Matrigel containing 30 mM Na-Lactate. There was a statistically significant difference between the growth profiles of 4T1-HRE A5NC versus A2-10KD animals (p = 0.03), but not between 4T1 HRE-A2-10KD in Matrigel and in Matrigel containing 30 mM Na-Lactate (**A**). Kaplan-Meyer survival of the three animal cohorts (**B**). Metastases formation after surgical resection of the primary 4T1-HRE A2-10KD tumors was assessed by bioluminescence imaging (BLI): a comparison of the number of metastases/mouse observed in the BLI images on days 4, 11, 18 and 22 after surgical resection of the primary tumor (**C**), and BLI images obtained on Day 22 (**D**). Five animals per group, one A2-10KD tumor-bearing animal died on day 21. Statistics: * p<0.05; ** p<0.01.

To determine whether the presence of exogenous Na-Lactate changes the metastatic profile of orthotopic 4T1 tumors, a separate group of animals (n = 5/group) was studied. 4T1-HRE A5NC (2 x 10^5^ tumors cells + matrigel) and A2-10KD control tumors (2 x 10^5^ tumor cells + matrigel) and test tumors (2 x 10^5^ tumor cells + matrigel + 30 mM Na-Lactate) were inoculated in immunocompetent mice. The primary orthotopic mammary tumors were surgically removed when tumors reached a volume of ~200 mm^3^. As expected, the control 4T1-HRE A5NC tumors proliferated faster than the A2-10KD (LDH-A KD) tumors (p = 0.03) (**Fig 5A**). A small enhancement of Na-Lactate on primary LDH-A KD tumor growth was detected, but did not reach statistical significance (**Fig 5A**). The 4T1-HRE A5NC tumor-resected animals (in matrigel alone) died first (30–33 days after tumor cell inoculation), with a median survival 31 days. Animals bearing resected LDH-A KD tumors (in Na-Lactate + matrigel plug) began to die shortly thereafter (37–48 days after tumor inoculation) (**Fig 5B**), with a median survival of 38 days. In contrast, animals bearing resected LDH-A KD tumors (in matrigel alone) survived longest (median survival 50 days)(**Fig 5B**).

In addition to overall survival, a notably greater number of metastases were detected by BLI in mice bearing LDH-A KD tumors with matrigel + Na-Lactate compared to controls with a matrigel alone (**Fig 5C and 5D**). By day 22 after surgery, only 2 of 4 mice in the control group (matrigel alone) showed small metastases in the lung area, while all mice in the test group (matrigel + Na-Lactate) showed 2 or more large lesions in different areas of the body (**Fig 5D**). These results demonstrated that the addition of Na-Lactate to the matrigel plug facilitated the development of tumor metastases.

### Clinical correlates

**Fig 6:**
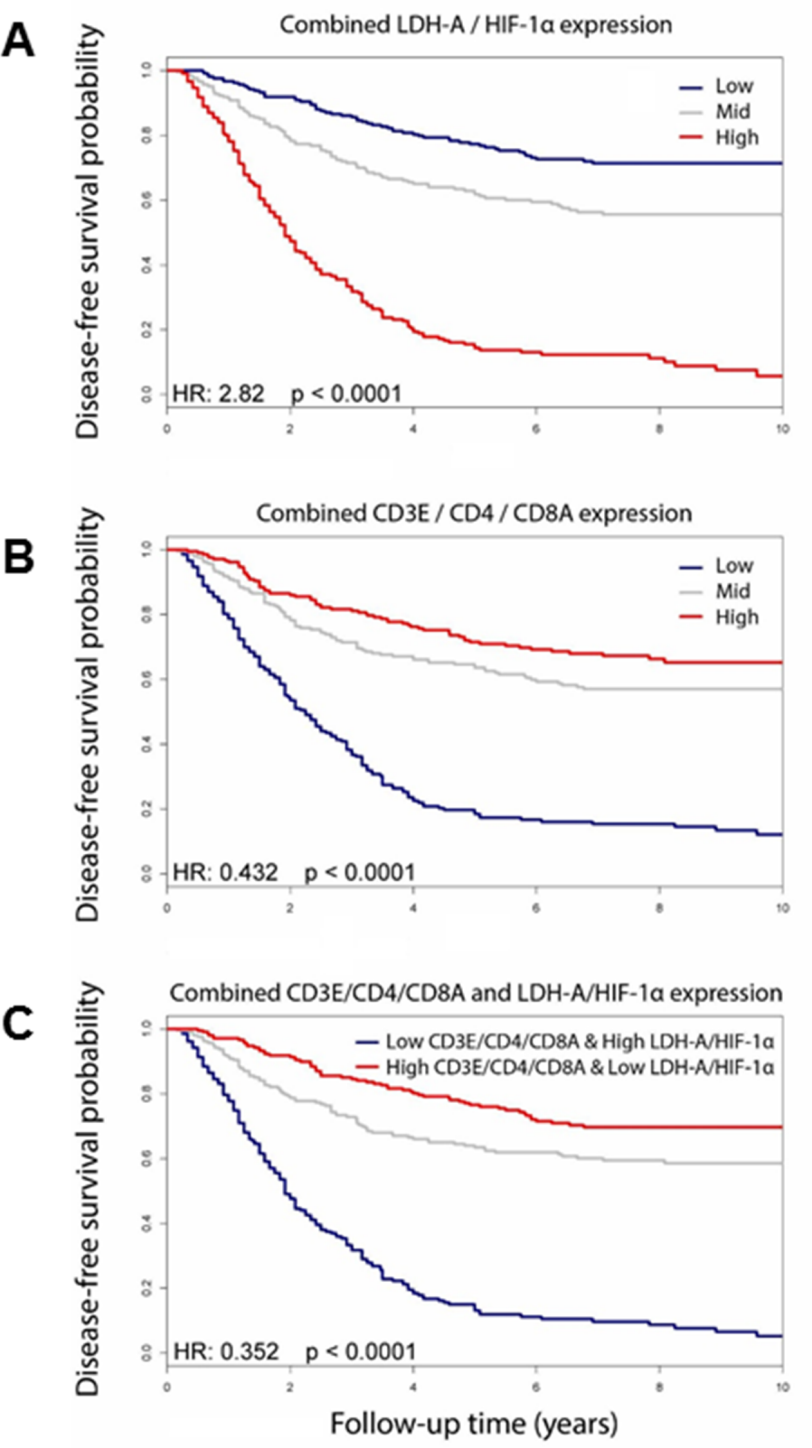
Kaplan-Meier estimators for metastasis-free survival. From a compendium of four breast cancer datasets (from GEO: GSE-2603, GSE2034, GSE5327, GSE12276). The combined expression of LDH-A and HIF-1α (**A**) and CD3E, CD4 and CD8A (**B**) was calculated for each patient, and the data set separated into thirds based on the combined expression levels, and Kaplan-Meier estimators and Cox proportional hazard ratios were calculated for the Low vs. High groups. The combined LDH-A and HIF1α expression was subtracted from the combined CD3E, CD4 and CD8A expression level for each patient; the patient data set was separated into thirds based on the “Combined CD3E/CD4/CD8A/LDH-A/HIF-1α” signature, and Kaplan-Meier estimators were calculated (**C**).

Our experimental results in a murine breast cancer model are consistent with a gene-expression analysis from a compendium of four human breast cancer datasets (from GEO: GSE-2603, GSE2034, GSE5327, GSE12276). There was a clear association between high LDH-A and HIF-1α expression and poor outcome (metastases-free survival); the Kaplan-Meir estimators were significantly different (p<10^−16^)—**Fig 6A**), indicating longer survival for patients with low expression of LDH-A and HIF-1α compared to the high expression group.

The opposite relationship was observed in patients with high expression of immune-related genes; high expression of CD3E, CD4 and CD8A was associated with a comparatively good outcome (p<10^−16^—**Fig 6B**) [41]. Not surprisingly, patients with poor outcomes had both high LDH-A/HIF-1α and low CD3E/CD4/CD8A levels of expression; whereas patients with good outcomes had both low LDH-A/HIF-1α and high CD3E/CD4/CD8A levels of expression (**Fig 6C**) [41].

## Discussion

LDH-A/lactate levels have a significant impact on the tumor microenvironment, disease evolution, progression, and development of metastases [1, 2, 11, 42–47]. Tumors with high tissue lactate concentrations and high LDH-A expression have been linked to poor prognosis [48–51], and are associated with greater metastatic potential [50, 51]. The downregulation of LDH-A in different tumor types [2–4, 8, 52] leads to an anti-proliferative effect on primary tumors and delays the development and progression of metastases [11, 53, 54].

Building on our previous studies that show LDH-A knockdown reduces orthotopic 4T1 breast tumor lactate in nude mice [11, 14, 55], we show a more robust anti-tumor effect in immune competent BALB/c mice, that includes significant changes in the tumor microenvironment (TME). Furthermore, to simulate the clinical management of breast cancer, we removed the primary tumors when they were relatively small (∼100–200 mm^3^) and reached similar sizes, and monitored the animals for the development of metastases and survival. We observed significantly improved metastasis-free and overall survival of mice in the LDH-A KD tumor-bearing group, with 40% long-term survivors. We attribute the development of fewer metastases and the improvement in overall survival of LDH-A KD group compared to control NC-tumor-bearing mice to changes in the TME. The observed reductions in HIF-1 activity and VEGF-A secretion observed in LDH-A KD tumor cells *in vitro*, translates to phenotypic changes of the TME *in vivo*. Others have shown that tumor vessel “normalization” is associated with CD4^+^ T cell infiltration (reflecting vascular and immune reprogramming), and results in fewer metastases and a better survival [56]. We identified reduced neo-vascularization, decreased necrosis, as well as a significant reduction in HIF-1α expression and pimonidazole staining, and a substantial enhancement of CD3^+^ and CD4^+^ infiltration of T-cells and reduction in F4/80^+^ TAMs in LDH-A KD compared to NC 4T1 tumors.

To study the association between LDH-A expression and HIF-1 activity, we developed 4T1-HRE A5NC and LDH-A KD cell lines that included: i) a HRE-responsive exGLuc reporter to monitor HIF-1 activity and ii) a constitutively expressed FLuc reporter to monitor the development of metastases. We detected a reduction of HIF-1 activity in LDH-A-depleted cells and this was confirmed by the observed reduction of VEGF-A secretion and other HIF-1 downstream targets. These data are consistent with the concept that lactate can trigger activation of HIF-1 in tumor cells under normal oxygen conditions and with a high levels of oxidative phosphorylation (OXPHOS)[19]. Lactate is known to function as a signaling molecule and a metabolic fuel [12, 26, 57, 58]. Previous studies have shown that the metastatic proclivity of 4T1 cells is associated with altered glycolysis, pentose phosphate pathway activity and fatty acid synthesis, as well as a decreased GSH/GSSG redox pool and an enrichment of tricarboxylic acid (TCA cycle) intermediates [59]. Interestingly, we also observed that 4T1 cells consume considerable amounts oxygen, demonstrating high mitochondrial respiration [14], which is not the traditional Warburg phenotype, and reflects the high metabolic plasticity of 4T1 cells [55]. Furthermore, a prominent paracrine control of HIF-1 by lactate was detected in aggressive metastatic 4T1-HRE A5NC control cells, notably more than in LDH-A knock-down cells. This was confirmed later by HRE-BLI results in primary tumors, HIF-1α and pimonidazole immunofluorescence imaging (**Fig 3**).

Since lactate directly promotes HIF-1 activity and VEGF-A secretion, we sought to determine whether the addition of lactate directly to the TME would affect tumor phenotype. Tumor LDH-A KD cells were inoculated in Matrigel plugs—in the presence or absence of 30 mM Na-Lactate. We detected a small increase in the rate of primary tumor growth of both LDH-A KD tumors (A2-10KD > A3-8KD), when inoculated in Matrigel + Na-Lactate, compared to Matrigel alone. More important was the greater number of metastases observed in mice bearing LDH-A KD tumors in the Matrigel + Na-Lactate group compared to the Matrigel-alone group. These data clearly demonstrate that the presence of Na-Lactate in the TME facilitates the development of metastases.

Moreover, we observed significantly less necrosis in small (100–200 mm^3^) LDH-A KD tumors compared to control NC tumors. The process of necrosis attracts macrophages into the tumor mass, leading to macrophage augmentation and modification of tissue homeostasis [60]. Tumor lactate (a product of LDH-A function) has an important signaling role in the polarization of TAMs, and in the subsequent promotion of tumor growth by the lactate-induced stabilization of HIF-1α and expression of its downstream target arginase 1 [60]. We observed a greater number of TAMs in the more aggressive A5NC tumors compared to the LDH-A KD tumors. Tumor cells secrete lactate into the TME, which activates macrophage recruitment, induces their functional polarization into TAMs, and stimulates vascular endothelial growth factor (VEGF) production in TAMs through HIF-1α [60]. We show an association between the density of TAMs and the degree of vascularization.

In addition to the role of TAMs and other stromal cells in tumor VEGF production, Judah Folkman pioneered the concept that tumors cells themselves can secrete VEGF. This secretion depends on HIF-1 activated pathways, stimulating both vascular endothelial cell proliferation and angiogenesis [61]. There are metabolic effects on VEGF production as well, since it has been shown that lactate and pyruvate, as end products of glycolysis, can regulate hypoxia-inducible gene expression independent of hypoxia [18]. Our observations of decreased HIF-1 read-out, downregulation of VEGF-A production and other HIF-1 targets in LDH-A depleted cells, are consistent with these findings. HIF-1 is highly expressed in many aggressive primary tumors and is associated with the development of metastasis in patients [62, 63]. HIF-driven metastases can occur through different mechanisms, and have an impact on each step of the metastatic cascade (invasion, migration, intravasation and extravasation). Hypoxia itself and changes in tumor metabolism through other molecular and cellular mediators impact the TME, the development of necrosis [64], and the production of VEGF-A [65].

What may be more important are factors that regulate the ability of tumor cells to evade immune attack [27], promoting the immunosuppressive phenotypes [66, 67]. The delayed appearance of metastases and prolonged survival in LDH-A KD tumor-bearing mice indicated that the microenvironment can play an important role in tumor progression and metastasis formation [68–70]. The aggressive tumor phenotype we observed in the 4T1 murine model is also observed clinically; it is associated with poor patient outcome [71, 72], and is independent of tumor size and stage [73, 74]. In a compendium of four human breast cancer datasets [75–78], we show a clear association between high LDH-A and HIF-1α gene expression and poor outcome, and an inverse pattern of disease-free survival with immune-related gene expression (CD3E/CD4 and CD8A). Most patients with poor outcomes had both high LDH-A/HIF-1α and low CD3E/CD8A/CD4 levels of expression. This indicates that lactate/hypoxia, and angiogenesis/VEGF-A are critical in promoting metastasis, as well as preventing the immune system from mounting a significant anti-tumor response in both untreated as well as treated patients. An unbiased analysis of 12 gene expression data sets (including the cohort described here, as well as the METABRIC and TCGA studies, among others) is currently being performed to identify a reliable set of glycolysis and immune signature genes that can be applied to breast and other cancers.

## Conclusions

Our studies in the 4T1 murine model of aggressive/metastatic breast cancer provide direct evidence that tumor cell metabolism (high LDH-A expression) is associated with a “poor prognosis” phenotype, and is mediated through more robust HIF-1α and downstream target responses to hypoxia. The link between tumor metabolism, the TME and the immune response (in an immune competent host) is complex and is supported by these observations. Furthermore, in patients with breast cancer, a limited bioinformatics analysis shows that patient outcome (metastases-free survival) is associated with an inverse pattern of metabolism-related (LDH-A and HIF-1α) and immune-associated (CD3E and CD8A, CD4) gene expression.

## Supporting information

S1 Fig The development 4T1-HRE-exGLuc-IRES2-GFP_tdRFP/FLuc cells. Murine breast cancer cells (4T1) were transduced with a retroviral vector bearing exGaussia Luciferase and GFP under hypoxia response element (HRE) HIF-1 activated promoter (**1**). Transduced 4T1 cells were selected with 500 μg/ml G418 and underwent a second transduction with tdRFP/FLuc dual reporters (**2**) (**A**). Cells were incubated under 1% oxygen for 24 h and sorted for a double positive GFP and tdRFP cells three times. The selected and sorted population of cells was assessed for bioluminescence reporter expression and activity under different conditions (**B**).
(TIF)

S2 Fig Characterization of 4T1-HRE LDH-A knockdown cells. Western blot analyses on 4T1 whole cell lysates prepared from 4T1-HRE A5NC and LDH-A KD clones (**A**). Total LDH enzyme activity in A5NC and LDH-A KD cells. Cells were grown in DMEM with 25 mM glucose, 6 mM L-glutamine and 10% FCS (A2-10KD<0.0001; A3-8KD = 0.0024)(**B**). *In vitro* cell growth over 5 days (**C**). The comparison of a Compensatory Glycolysis between control and LDH-A knock-down cells, ** p<0.01;**** p<0.0001 (**D**, **E**).
(TIF)

S3 Fig LDH-A depletion: effect on intra-tumoral host cells properties. Comparison of density of CD4^+^ T cells was assessed and a trend to higher numbers was detected in LDH-A knock-down cells (**A).** Profile of CD3^+^ T cells fluorescence (from tumor periphery to center) of A5NC (control) and LDH-A KD tumors (**B**). We also noticed that CD3^+^ T cell size increased in LDH-A KD tumors from 50.7±3.9 μm^2^ in control to 61±2 μm^2^ in LDH-A knock-down tumors, p<0.0001 (**C**). All tumors (4T1-HRE A5NC and LDH-A KD) were divided into small regions of interest and the percentage of CD3- and CD31-positive pixels was calculated and plotted, revealing an inverse relationship between CD3+ T cells and CD31+ tumor vascularity (**D**).
(TIF)

S4 Fig Impact of hypoxia and LDH-A knockdown on HIF-1 downstream gene expression. HK1, HK2 expression was evaluated by Western blotting in 4T1-HRE cell lines: A2-10KD (LDH-A shRNA knockdown) and A5-NC (scrambled shRNA control) after 6 h of normoxic and hypoxic growth conditions. The bar graph columns correspond to the Western blots above (**A**). LDH-A, MCT1, MCT4 mRNA levels were evaluated by ddPCR (**B**).
(TIF)

S5 Fig Lactate effect (matrigel plug) on LDH-A KD tumor growth. A comparison of orthotopic tumor cell implantation with matrigel alone or matrigel with 30 mM Na-Lactate is shown. Tumor growth profiles following orthotopic injection of 2x10^5^ LDH-A knock-down cells (4T1 HRE-A2-10KD (**A**) and 4T1HRE-A3-8KD (**B**)).
(TIF)
