# Supplementary figures and images for: LDH-A regulates the tumor microenvironment via HIF-signaling and modulates the immune response
Source: PLoS One. 2018 Sep 24;13(9):e0203965. doi: 10.1371/journal.pone.0203965 (PMC6153000; doi:10.1371/journal.pone.0203965)

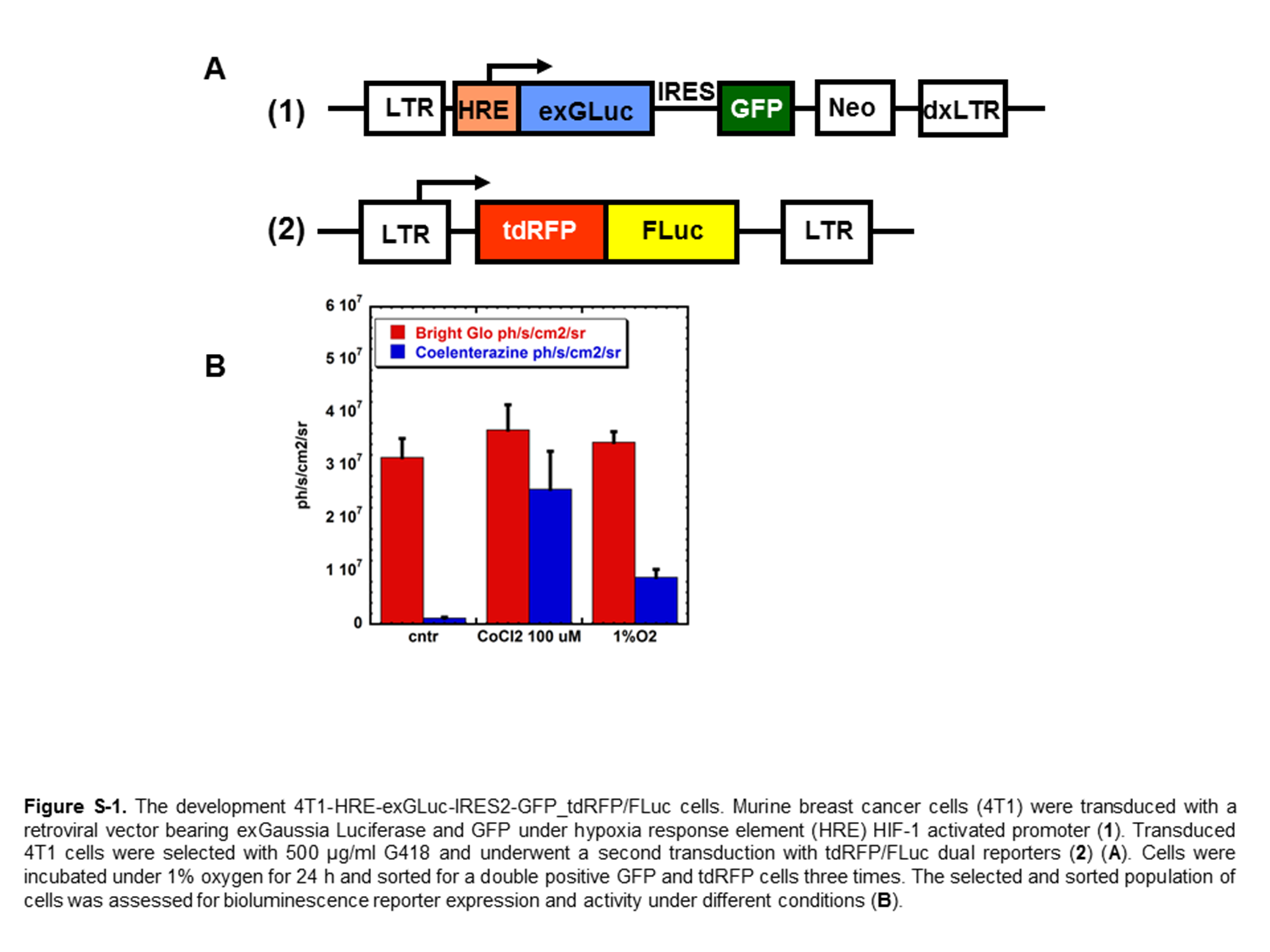

Supplement: S1 Fig — Murine breast cancer cells (4T1) were transduced with a retroviral vector bearing exGaussia Luciferase and GFP under hypoxia response element (HRE) HIF-1 activated promoter (1). Transduced 4T1 cells were selected with 500 μg/ml G418 and underwent a second transduction with tdRFP/FLuc dual reporters (2) (A). Cells were incubated under 1% oxygen for 24 h and sorted for a double positive GFP and tdRFP cells three times. The selected and sorted population of cells was assessed for bioluminescence reporter expression and activity under different conditions (B). (TIF) [file pone.0203965.s001.tif]

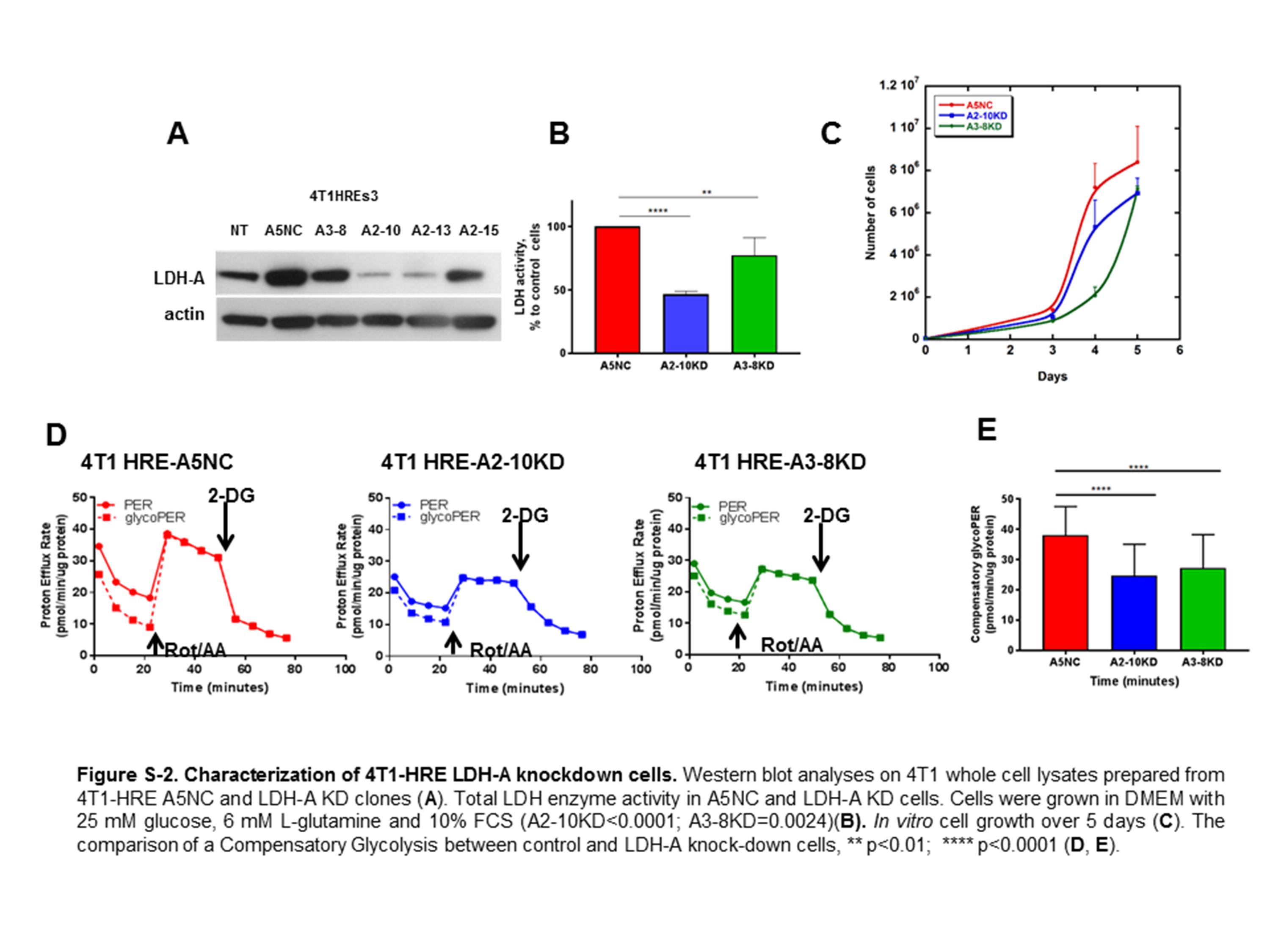

Supplement: S2 Fig — Western blot analyses on 4T1 whole cell lysates prepared from 4T1-HRE A5NC and LDH-A KD clones (A). Total LDH enzyme activity in A5NC and LDH-A KD cells. Cells were grown in DMEM with 25 mM glucose, 6 mM L-glutamine and 10% FCS (A2-10KD<0.0001; A3-8KD = 0.0024)(B). In vitro cell growth over 5 days (C). The comparison of a Compensatory Glycolysis between control and LDH-A knock-down cells, ** p<0.01;**** p<0.0001 (D, E). (TIF) [file pone.0203965.s002.tif]

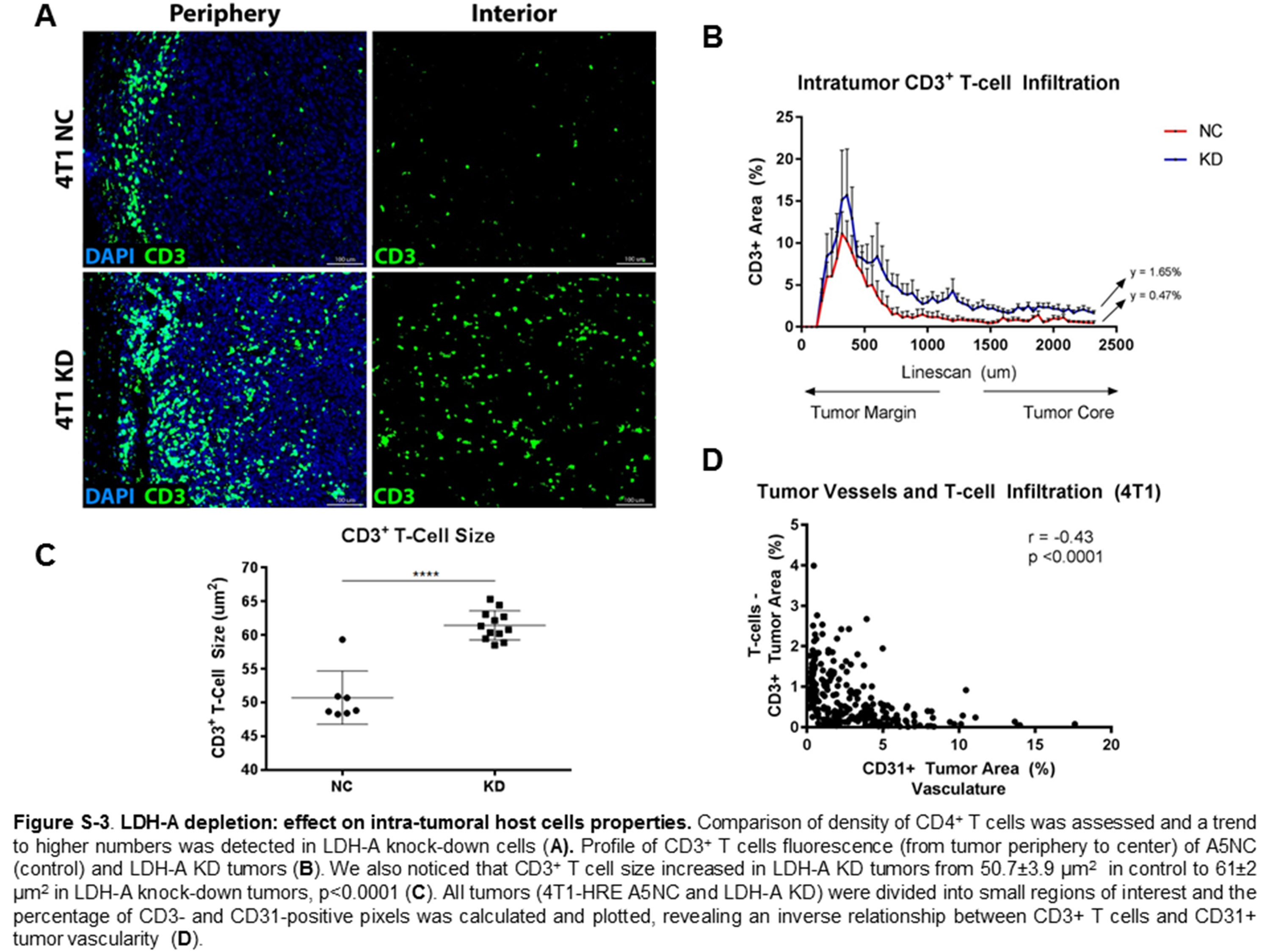

Supplement: S3 Fig — Comparison of density of CD4+ T cells was assessed and a trend to higher numbers was detected in LDH-A knock-down cells (A). Profile of CD3+ T cells fluorescence (from tumor periphery to center) of A5NC (control) and LDH-A KD tumors (B). We also noticed that CD3+ T cell size increased in LDH-A KD tumors from 50.7±3.9 μm2 in control to 61±2 μm2 in LDH-A knock-down tumors, p<0.0001 (C). All tumors (4T1-HRE A5NC and LDH-A KD) were divided into small regions of interest and the percentage of CD3- and CD31-positive pixels was calculated and plotted, revealing an inverse relationship between CD3+ T cells and CD31+ tumor vascularity (D). (TIF) [file pone.0203965.s003.tif]

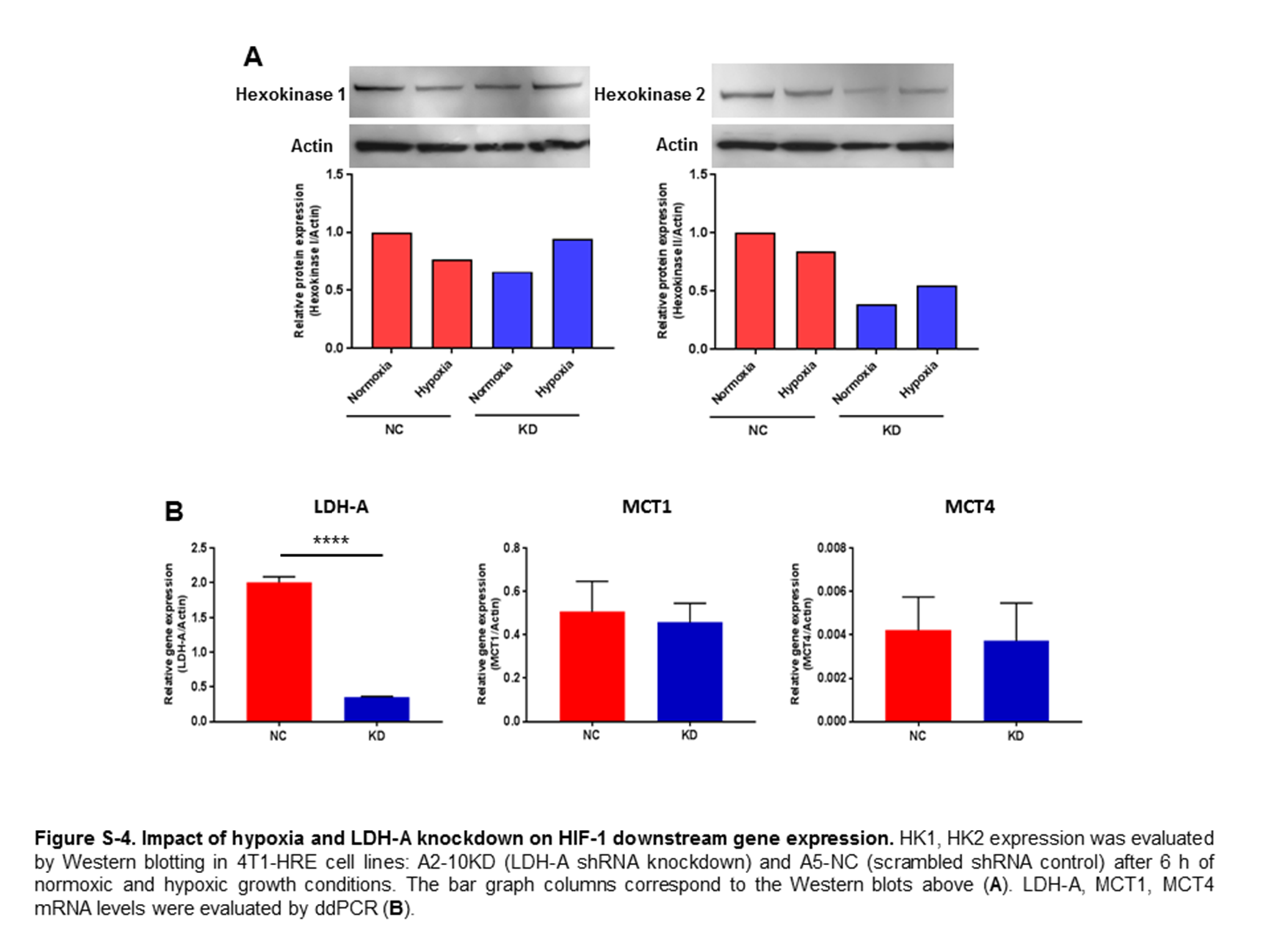

Supplement: S4 Fig — HK1, HK2 expression was evaluated by Western blotting in 4T1-HRE cell lines: A2-10KD (LDH-A shRNA knockdown) and A5-NC (scrambled shRNA control) after 6 h of normoxic and hypoxic growth conditions. The bar graph columns correspond to the Western blots above (A). LDH-A, MCT1, MCT4 mRNA levels were evaluated by ddPCR (B). (TIF) [file pone.0203965.s004.tif]

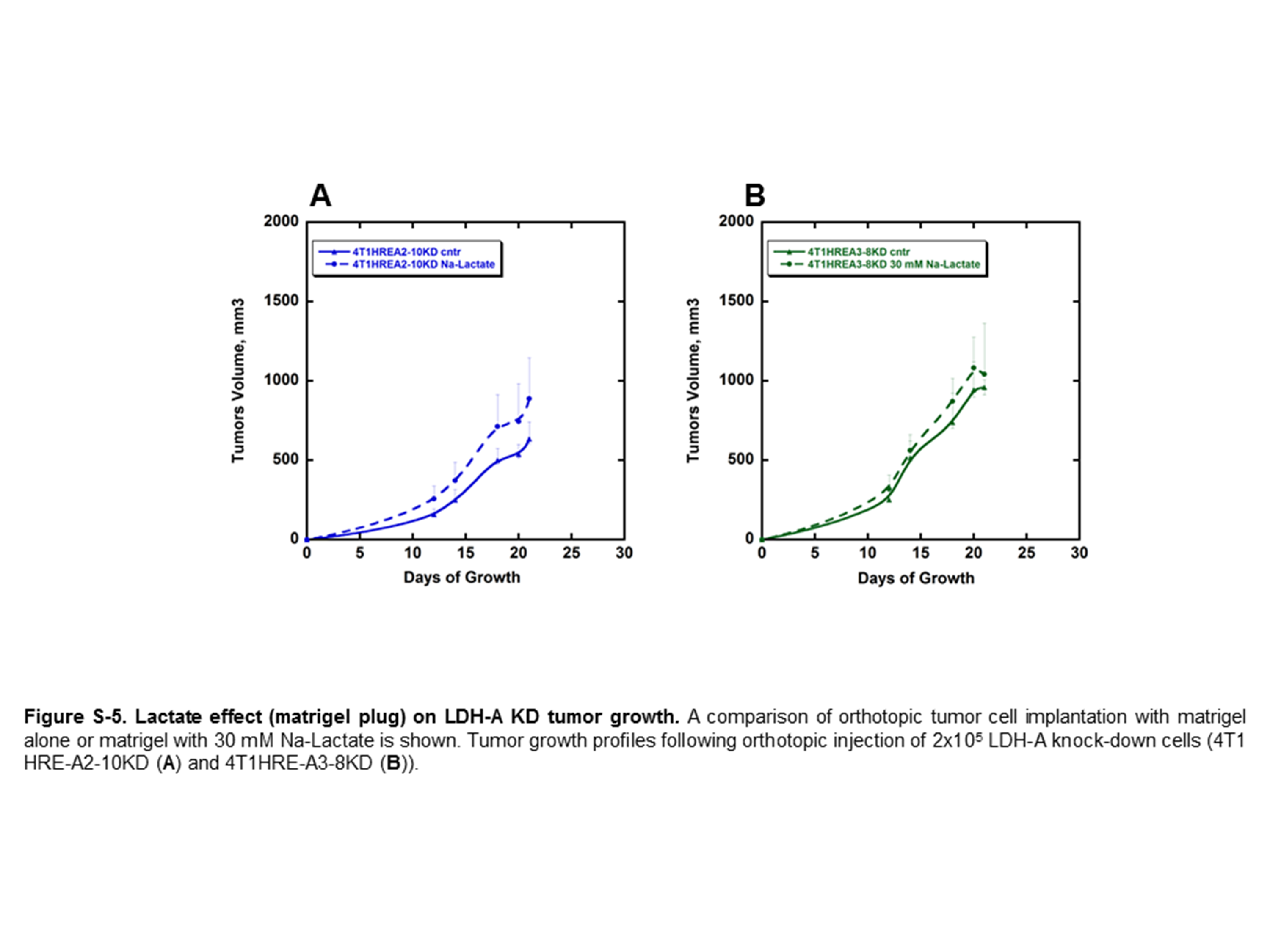

Supplement: S5 Fig — A comparison of orthotopic tumor cell implantation with matrigel alone or matrigel with 30 mM Na-Lactate is shown. Tumor growth profiles following orthotopic injection of 2x10^5^ LDH-A knock-down cells (4T1 HRE-A2-10KD (A) and 4T1HRE-A3-8KD (B)). (TIF) [file pone.0203965.s005.tif]
